# Impact of titanium dioxide nanoparticles on purification and contamination of nematic liquid crystals

**DOI:** 10.3762/bjnano.8.275

**Published:** 2017-12-21

**Authors:** Dmitrii Pavlovich Shcherbinin, Elena A Konshina

**Affiliations:** 1 Department of Optical Physics and Modern Natural Science, ITMO University, Kronverkskiy pr. 49, Saint Petersburg 197101, Russia

**Keywords:** ionic impurities, liquid crystals, nanoparticles, titanium dioxide

## Abstract

We have investigated the impact of titanium dioxide nanoparticles on the ionic contamination of liquid crystals. Nematic liquid crystals with high and low initial ionic contamination have been examined. It has been shown that titanium dioxide nanoparticles reduced the ion density of liquid crystals with high initial ionic contamination from 134.5 × 10^12^ cm^−3^ to 63.2 × 10^12^ cm^−3^. In the case of liquid crystals with low initial ionic contamination, the nanoparticles led to an insignificant increase of ion density from 19.8 × 10^12^ cm^−3^ to 25.7 × 10^12^ cm^−3^.

## Findings

Nowadays, liquid crystals (LCs) are widely used in different display and non-display applications. The development of display techniques has led to the construction of high-quality screens including fully transparent [1], flexible screens [2] and dual-view [3] displays in the past years. The area of LCs for non-display applications is also rapidly growing. The application of LCs includes metamaterials [4], photonic crystals [5], plasmonic structures [6], THz devices [7], sensors [8], diffractive optics [9], adaptive lens technologies [10] and vision correction [11], as well as tunable filters [12] and dispersion for imaging [13]. In addition, nanoparticles can induce other new functions in liquid crystals, including improved response time [14–15], surface plasmon resonance [16], and improvements in alignment [17].

The ionic contamination of LCs remains one of the challenges to LC technology. Ionic conductivity negatively affects LC device performance, leading to slow response, a reduction of the voltage holding ratio, and image quality degradation. Despite the negative effect of ionic contamination in display techniques, LCs with high ionic conductivity may be used in non-display applications [18–19]. Although modern LC mixtures are highly purified, uncontrolled contamination during utilization can occur [20]. Physical–chemical methods provide a desirable level of LC purity during synthesis which cannot be applied during the process of using the LC devices. The search for a new solution to control the ionic contamination induced during LC device utilization is a current research focus in the field of LC display techniques.

Extensive experimental research has shown that nanoparticles (NPs) affect the ionic conductivity of LCs. However, data obtained from different experimental studies are contradictory. The decrease of ionic contamination was observed in LCs doped with carbon nanoparticles [21], semiconductor quantum dots [22], and metal NPs [23]. At the same time, the increase of ionic contamination due to doping with NPs was reported in other papers [24–26]. This contradiction was resolved by the theory developed by Garbovskiy et al. [27–30]. In the framework of this theory it was shown that the same nanoparticles can lead to both contamination and purification of LCs. The theory considers adsorption and desorption of ions on nanoparticle surfaces and takes into account the initial ionic contamination of LCs. The same NP can lead to both purification and contamination of LCs under certain conditions. The aim of this study is the experimental verification of Garbovskiy’s assumption that the same NPs may have a different effect on the LC ionic conductivity depending on the initial LC contamination. We have examined nematic liquid crystals with low (LC1) and high (LC2) ionic contamination and their composites with TiO_2_ nanoparticles. The impact of TiO_2_ nanoparticles on purification and contamination in LCs has been experimentally shown.

The plane-parallel cells consisted of two glass substrates covered by indium-tin oxide (ITO) electrodes and rubbed polyimide layers were used for measurements. The thickness of the LC cells was set by spacers and controlled by measurements of empty cell capacitance. The thickness of the cells was 15 ± 1 μm. We used commercial nematic LC (ZhK1282, NIOPIK, Moscow) with low initial ionic contamination (LC1). The ionic surfactant cetyltrimethylammonium bromide (CTABr) was added to the same LC to produce a LC with high ionic conductivity (LC2). The surfactant dissolves in LCs and dissociates on Br^−^ and CTA^+^ ions [31]. The concentration of CTABr in LC2 was about 0.1 wt %. LC2 was studied as a sample of LC that was contaminated during the utilization process. Titanium dioxide nanoparticles (Plasmotherm, Moscow) were doped into LC1 and LC2. The nanoparticles were a mixture of anatase and rutile with an average particle size of 50 nm. Used NPs were obtained by plasma synthesis and were not covered with any ligands. Dry NPs were added to LCs at a concentration of 0.25, 0.5 and 1 wt %. The composites were prepared in isotropic phase over one hour by ultrasonication. To estimate ion density (*c*) and average diffusion coefficient (*D*) in LC1, LC2 and their composites with TiO_2_ NPs, low frequency dielectric spectra were measured. All measurements were performed with a LCR-meter (Agilent E4980A) under the same conditions at room temperature.

**Figure 1:**
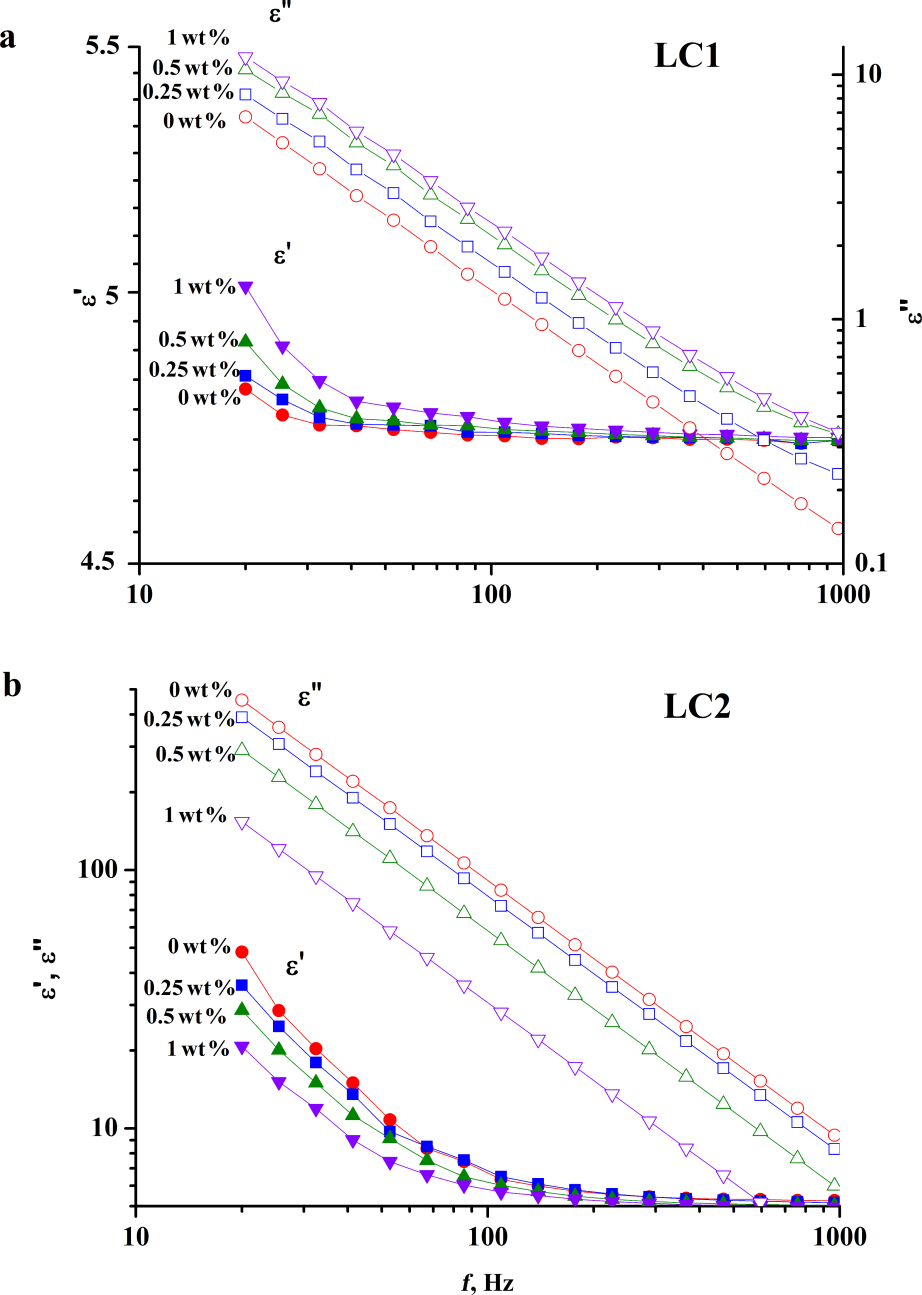
The low frequency spectra of the real and imaginary parts of the dielectric permittivity for LC1 (a), LC2 (b), and their composites.

The investigation results of the low frequency dielectric spectra LCs are shown in Figure 1. The spectra were obtained in the range from 20 Hz to 1 kHz. In this spectral range, the dispersion of dielectric permittivity is related to ionic conductivity. Figure 1a represents real and imaginary parts of the dielectric permittivity of initially low-contaminated liquid crystals (LC1) and their composites with TiO_2_ nanoparticles. The increase of NP concentration led to the enhancement of the real and imaginary parts of the permittivity. We have observed an insignificant change in the spectra shown in Figure 1a. In contrast, the changes in the spectra were significant in the case of the initially high-contaminated LC2 (Figure 1b). Doping LC2 with TiO_2_ NPs resulted in a reduction of the real and imaginary parts of the dielectric permittivity. This indicates a decrease of the ionic conductivity of LC2.

In this frequency range, the spectra of the dielectric permittivity can be approximated by following equations [24]:


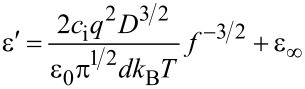



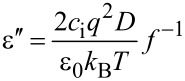


where *с*_i_ – ion density, *q* – elementary charge, *D* – average diffusion coefficient, ε_0_ – dielectric constant, *d* – thickness of the cell gap, *k*_B_ – Boltzmann factor, *T* – temperature, *f* – frequency, and ε_∞_ – high-frequency dielectric permittivity.

We have evaluated the ion density and the average diffusion coefficient of LC1, LC2 and their composites. The data are shown in Table 1.

**Table 1:** Ion density and average diffusion coefficient of LC1, LC2 and their composites.

TiO_2_ concentration, wt %	LC1		LC2	
	
	Ion density × 10^12^ cm^−3^	Diffusion coefficient × 10^−8^ cm^2^/s	Ion density × 10^12^ cm^−3^	Diffusion coefficient × 10^−8^ cm^2^/s

0	19.8	3.8	134.5	47.5
0.25	23.2	4.2	105.2	52.3
0.5	24.9	5.6	72.7	56.2
1	25.7	6.5	63.2	60.0

The initial data show that increasing the TiO_2_ NP concentration up to 1 wt % leads to the rise of the ion density to 30% in the case of initially low-contaminated LC1. In the case of initially high-contaminated LC2, the ion concentration is reduced almost twice by doping with 1 wt % TiO_2_ NPs. In both cases, the increase of concentration leads to an increasing diffusion coefficient. Moreover, the estimated diffusion coefficient of ions in LC2 was larger.

In the framework of Garbovskiy’s theory, the surface of the NP is considered as a surface with absorbing sites. Because of adsorption/desorption processes on adsorbing sites, the concentration of ions in the LC composites change. Ideal nanoparticles will have a high surface density of adsorbing sites which are completely unoccupied. This ideal NP surface would result in more pure LCs, regardless of the initial ionic contamination. However, the adsorbing sites of real NPs are partially occupied. In general, the effect of doping LCs with NPs on the ionic conductivity depends on the initial contamination of the LC as well as the surfaces states of the NPs, ion adsorption and desorption rates. Adsorption/desorption rates and contamination of the NP surface define a critical concentration of ions. If the initial concentration of mobile ions in LCs is beyond the critical concentration, doping the LC with NPs will lead to purification of the LC. If the initial concentration of mobile ions in a LC is lower than the critical concentration, doping the LC with NPs will lead to contamination of the LC. In our experiments, TiO_2_ NPs purified LC2 with an initial ion concentration of about 10^14^ cm^−3^ and slightly contaminated LC1 with an initial ion concentration of about 10^13^ cm^−3^ (Table 1).

There are several types of ions in liquid crystals in our experiments such as the ions present in the initial LC, ions adsorbed on the surfaces of nanoparticles, ionic impurities introduced by aligning layers, glue, and Br^−^ and CTA^+^ ions in the case of LC2. All of these types of ions may have different density, size, diffusion coefficient and adsorption/desorption rates. In such complicated systems, the proportion between different types of ions can change [24,29] and may influence the average diffusion coefficient. Moreover, the change of dopant concentration may have resulted in the variation of the activation energy that affected the diffusion coefficients [32].

To conclude, we would like mention other factors that influence the ionic conductivity of the LC composites. As it was mentioned above, a high density of unoccupied adsorbing states is desirable. Size, aggregation, local fields near nanoparticles and the action of a high electric field affect the purification/contamination processes. NPs with smaller size have a larger surface area thereby facilitate purification/contamination processes. Nanoparticles in LCs tend to aggregate. This leads to the reduction of the total number of adsorbing sites. Moreover, in composites with a higher NP concentration, the aggregation process is stronger. Dry nanoparticles tend to aggregate in a nematic liquid crystal matrix. We have observed this aggregation in a polarized optical microscope at a concentration of over 0.5 wt %. This imposes restrictions on the concentration of NPs.

Several studies [33–35] considered the local fields near nanoparticles as a trap of ions. Such a consideration is acceptable for NPs with high polarizability such as graphene or ferroelectric NPs. The next factor is a high electric field. It was shown that an electric field higher than 2.5 V/μm induces the desorption of ions [27].

We have studied the impact of TiO_2_ NPs on nematic LCs with different initial ionic contamination. It has been shown that NPs reduced the ionic density of LCs by two times with an initial contamination of 134.5 × 10^12^ cm^−3^. These types of NPs can be used to prevent uncontrolled ionic contamination that occurs during LC device production and utilization. The study of the nature of the adsorption states, the estimation of adsorption and desorption rates of ions for various types of NPs, will be the next step in understanding the effect of doping liquid crystals with nanoparticles and the content of ion impurities resulting from this process.
